# Heat shock drives genomic instability and phenotypic variations in yeast

**DOI:** 10.1186/s13568-020-01091-7

**Published:** 2020-08-17

**Authors:** Li Shen, Yu-Ting Wang, Xing-Xing Tang, Ke Zhang, Pin-Mei Wang, Yang Sui, Dao-Qiong Zheng

**Affiliations:** Ocean College, Zhejiang University, Zhoushan, 316021 Zhejiang China

**Keywords:** Yeast, Heat shock, Mitotic recombination, Aneuploidy, Phenotypic evolution

## Abstract

High temperature causes ubiquitous environmental stress to microorganisms, but studies have not fully explained whether and to what extent heat shock would affect genome stability. Hence, this study explored heat-shock-induced genomic alterations in the yeast *Saccharomyces cerevisiae*. Using genetic screening systems and customized single nucleotide polymorphism (SNP) microarrays, we found that heat shock (52 °C) for several minutes could heighten mitotic recombination by at least one order of magnitude. More than half of heat-shock-induced mitotic recombinations were likely to be initiated by DNA breaks in the S/G_2_ phase of the cell cycle. Chromosomal aberration, mainly trisomy, was elevated hundreds of times in heat-shock-treated cells than in untreated cells. Distinct chromosomal instability patterns were also observed between heat-treated and carbendazim-treated yeast cells. Finally, we demonstrated that heat shock stimulates fast phenotypic evolutions (such as tolerance to ethanol, vanillin, fluconazole, and tunicamycin) in the yeast population. This study not only provided novel insights into the effect of temperature fluctuations on genomic integrity but also developed a simple protocol to generate an aneuploidy mutant of yeast.

## Key points

- Heat shock stimulates mitotic recombination and aneuploidy events in yeast.
- Trisomy events outnumbers monosomy events in heat-shock-treated cells.
- Heat shock and carbendazim treatment led to different patterns of genomic alterations.
- Heat shock fuels phenotypic variations in yeast population.

## Introduction

The yeast species *Saccharomyces cerevisiae*, like most cold-blooded organisms, experiences constant environmental change. Normally, yeast cells show optimal growth within a short temperature range (25–30 °C) (Morano et al. 2012), and temperatures above 36 °C would trigger heat shock response in yeast cells and affect their normal physiological activities (Yamamoto et al. 2007; Morano et al. 2012; Caspeta et al. 2014). Understanding how *S. cerevisiae* responds to heat shock would not only enrich our knowledge of cell biology but would also provide references for developing robust strains for industrial application (Abdelbanat et al. 2010; Huang et al. 2018; Morard et al. 2019).

As a model organism, *S. cerevisiae* has been widely used to explore heat shock response modulators. Studies have reported that multiple biochemical factors contribute to yeast’s tolerance and cell viability under high-temperature conditions (Morano et al. 2012). First, high temperature can greatly induce heat shock proteins (HSPs) to prevent the formation of protein aggregates and to help proteins acquire their normal functions (Piper 1995; Morano et al. 2012). Second, heat-shock-treated cells would accumulate trehalose, which stabilizes proteins and membrane (Conlin and Nelson 2007). Mutant yeast strains that are deficient in trehalose synthesis have been found to be sensitive to high temperature (Conlin and Nelson 2007; Saleh et al. 2014). Finally, antioxidant factors (superoxide dismutase, catalase, glutathione, and thioredoxin) were required to scavenge heat-shock-induced reactive oxygen species (ROS) and maintain redox homeostasis (Yamamoto et al. 2007). While research has extensively explored heat-shock-induced physiological changes (Richter et al. 2010; Morano et al. 2012), the impact of heat shock on genome integrity was less clarified. In *Candida albicans*, it was revealed that heat shock (39 °C, 42 °C and 50 °C) would stimulate chromosomal aneuploidy and rearrangements (Bouchonville et al. 2009; Forche et al. 2011). However, the signature of heat-shock-induced chromosomal instability and whether similar results would be found in *S. cerevisiae* was not yet clear.

Our recent studies used the diploid *S. cerevisiae* strains produced by crossing two heterozygous haploid strains to determine genomic instability caused by DNA replication stress (Zheng et al. 2016; Sui et al. 2020), oxidative stress (Qi et al. 2019a, b; Zhang et al. 2019), and small molecular compounds (Qi et al. 2019a, b; Sheng et al. 2019). The abundant single nucleotide polymorphisms (SNPs) between the two homologs in the diploid strains allow for the identification of genetic events across the yeast genome at high resolution through SNP microarray and genome sequencing (Guo et al. 2017; Yin et al. 2017; Zheng and Petes 2018). In this study, heat-shock-induced genomic instability was investigated in a *S. cerevisiae* strain using genetic screening systems and customized SNP microarrays. Our findings enriched our knowledge of how temperature fluctuations affect genome integrity and phenotypic evolution.

## Materials and methods

### Yeast strains and medium

The *S. cerevisiae* strain JSC25-1 (*MAT*a*/MATα::HYG ade2-1/ade2-1 can1-100/can1 ura3-1/ura3 leu2-3,112/LEU2 his3-11,15/HIS3 trp1-1/TRP1 IV1510386::KANr-can1-100/IV1510386::SUP4-o GAL2/gal2*) is a diploid strain constructed by crossing of haploids isogenic to W303-1A and YJM789 (St Charles and Petes 2013). JSC25-1 is heterozygous for all SNPs that distinguish W303-1A and YJM789 except for a small region (coordinate 369,892–373,127) on chromosome (chr) XV, which is homozygous for YJM789-derived SNPs. The diploid strain JSC24-2, which has the same background as JSC25-1, was used as a control in the SNP microarray analysis (St Charles and Petes 2013). The YPD medium contained 1% yeast extract, 2% peptone, and 2% glucose. To prepare solid plates, 2% agar was added into the medium, and to screen resistance mutants, YPD plates containing certain stressors (120 g/L ethanol, 0.1 g/L fluconazole, 1.2 g/L vanillin, and 4 mg/L tunicamycin) were prepared.

### Heat shock treatment and cell viability determination

JSC25-1 cells were grown in a 20 mL YPD liquid medium with an initial OD_600_ of 0.05 for 16 h. Cells were collected in PCR tubes (50 µL), heated at 52 °C for 2–4 min, and then plated on YPD plates (incubated for 3 days at 30 °C) to determine cell viability and the rate of sectored colonies.

### PCR diagnosis of the left-arm heterozygosity of chr IV

Compared with W303-1A-derived chr IV, YJM789 has a sequence deletion between 435,284 bp and 435,391 bp (*Saccharomyces* genome database (SGD) coordinates). Using a pair of primers (5′-AACCTTTAACATTCAGGGAG-3′ and 5′-ATGACTGCTTGGTAGTTGAG-3′) flanking the deletion, we can detect two DNA bands (404 bp and 296 bp) when the template DNA was heterozygous.

### Carbendazim treatment and mutant screening

A total of 40 JSC25-1 colonies formed on the YPD plate were picked and cultured independently in 5 mL YPD with 25 mg/L carbendazim (Adamas-beta, Shanghai, China) for 24 h at 30 °C (initial OD_600_ of 0.05). The cells from each culture were washed by ddH_2_O twice and plated on YPD plates independently to form colonies. Finally, 40 independent colonies after carbendazim treatment were selected for DNA extraction and SNP microarray analysis.

### Genomic DNA extraction and sonication

Yeast cells were cultured in 7 mL YPD media for 24 h with an initial OD_600_ of 0.05. For each strain, 60 mg cells were collected through centrifugation (5,000 rpm for 5 min) and embedded in low-melting agarose plugs with 1.2 mg/mL Zymolyase 20 T (Seikagaku, Tokyo, Japan). The plugs were incubated in a 1 mL buffer solution (500 mM EDTA, 10 mM Tris, pH 7.5) at 37 °C for 16 h. Proteinase K (2 mg/mL) (Sigma-Aldrich, MO, USA) was then added to the solution, and the plugs were incubated at 50 °C for 12 h. Finally, the plugs were washed in a 10 mL TE buffer (2 mM Tris, 1 mM EDTA, pH 8.0) at 4 °C for 48 h and a GeneJET PCR purification kit (Thermo Scientific, Waltham, MA, USA) was used to extract the DNA embedded in the plugs. Genomic DNA was then sonicated to fragments with an average size of 400 bp using a Bioruptor sonication device (Diagenode, Liège, Belgium).

### SNP microarray analysis

To explore how heat shock affects chromosomal stability, the whole-genome SNP microarray that can analyze ~ 13,000 SNPs across the yeast genome (Charles et al. 2012) and the chr IV-specific SNP microarray (St Charles and Petes 2013) were used to detect genetic alterations in JSC25-1-derived isolates. The SNP microarrays were designed based on the SNPs between the W303-1A and YJM789 genomes (Charles et al. 2012; St Charles and Petes 2013) and were produced by Agilent (Santa Clara, CA, USA). For each selected SNP, four 25-base oligonucleotides were used: two are specific to the W303-1A-derived SNP, and the other two are specific to the YJM789-derived SNP. The SNP was situated amid the 25-base oligonucleotide. In the SNP microarray experiment, the control DNA (200 ng) extracted from the JSC24-2 cells and experimental DNA (400 ng) were labeled dUTP-Cy3 and dUTP-Cy5, respectively, using the Invitrogen BioPrime array CGH labeling system (Thermo Scientific, Waltham, MA, USA). The labeled DNAs were purified using a GeneJET PCR purification kit (Thermo Scientific, Waltham, MA, USA) and then cohybridized onto microarray slides at 62 °C for 18 h. A GenePix 4000B scanner (Molecular Devices, Sunnyvale, CA, USA) was used to scan the slides, and GenePix 6.0 software (Molecular Devices, Sunnyvale, CA, USA) quantified the hybridization signals. The SNP microarray raw data was then entered in a GEO database (https://www.ncbi.nlm.nih.gov/geo/) with accession numbers GSE112062 and GSE150711.

### Stress tolerance test

The yeast isolates were incubated in 20 mL YPD and a YPD medium containing a certain stressor at 30 °C (or an indicated temperature) with an initial OD_600_ of 0.05. The stress conditions were 70 g/L ethanol, 0.15 mg/L fluconazole, 0.8 g/L vanillin, and 1.5 mg/L tunicamycin. The biomass formation (OD_600_) of yeast cells was detected using a spectrophotometer.

### Statistical analysis

Fisher’s exact test with a two-tailed *P* value and a *t*-test was conducted in VassarStat (https://vassarstats.net/). Two-way hierarchical clustering analysis of these aneuploidy events was performed using the R package “pheatmap” (Kolde and Kolde 2015).

## Results

### Heat shock stimulates genomic instability in *S. cerevisiae* JSC25-1

**Fig. 1:**
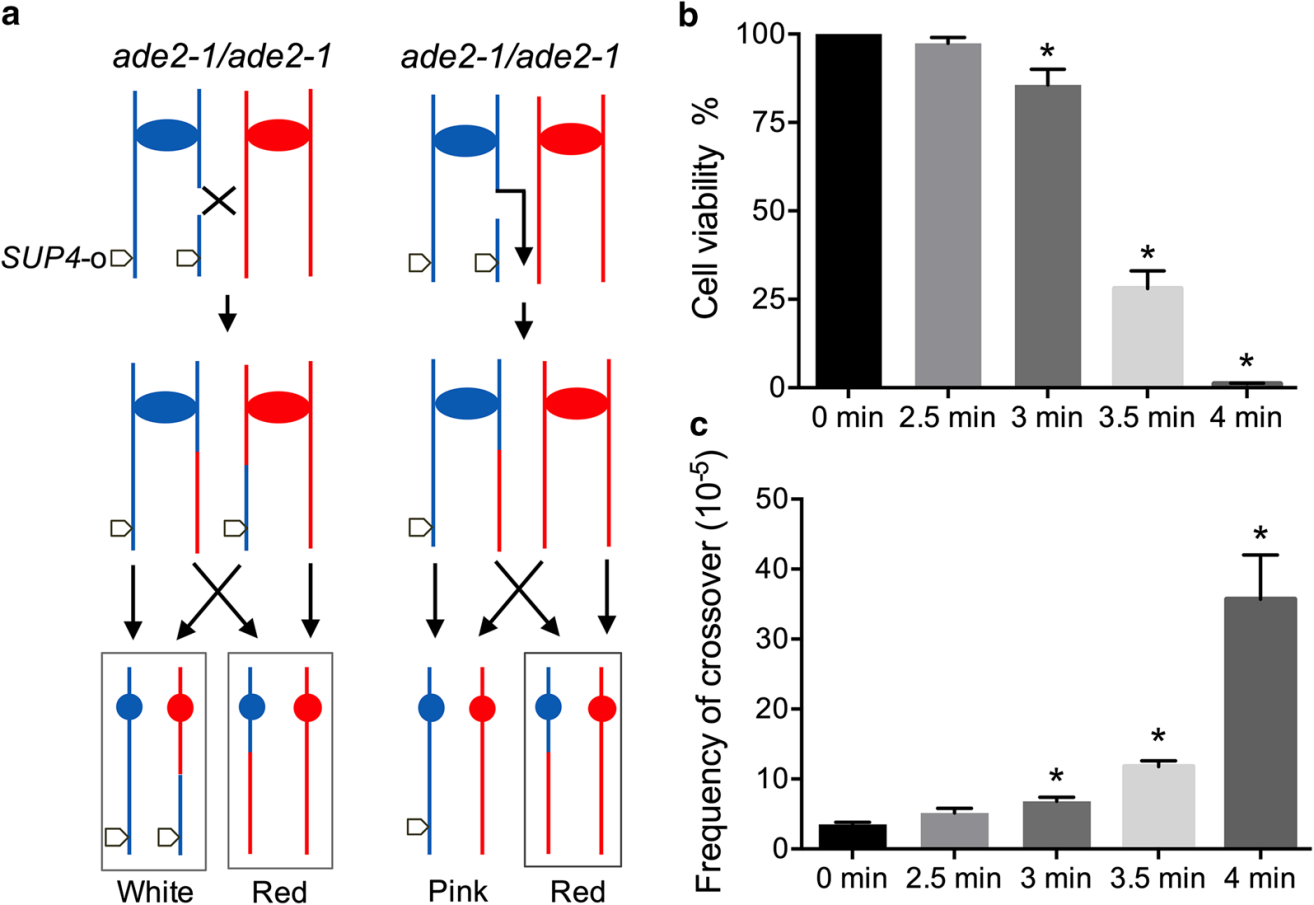
Heat shock stimulates mitotic recombination in the yeast strain JSC25-1. **a** A reciprocal crossover event on the right arm of chromosome IV would produce white/red-sectored JSC25-1 colonies. Alternatively, break-induced replication (nonreciprocal crossover) would produce white/pink- or red/pink-sectored colonies. **b** Cell viability of the JSC25-1 yeast strain after heat shock exposure (52 °C) for 2–4 min. **c** Reciprocal crossover frequency determined by calculating the red/white-sectored colonies formed on YPD plates after heat shock. “*” indicates significant difference at the level of 0.01 using *t*-test

To determine the effect of heat shock on chromosomal stability in *S. cerevisiae*, a sectored colony assay system was first used. *S. cerevisiae* JSC25-1 is homozygous for *ade2-1,* which displays a red phenotype because of a red pigment accumulation (a precursor of adenine) (St Charles and Petes 2013). In this strain, the ochre-suppressing tRNA mutant gene *SUP4-o* was inserted in the right end of the YJM789-derived chr IV (St Charles and Petes 2013). Since only one *SUP4-o* copy could partly suppress the ochre mutation of *ade2-1*, JSC25-1 formed pink colonies on the solid medium. Increased (two) and reduced (zero) *SUP4-o* copies in JSC25-1-derived isolates produce white and red colonies, respectively. Figure 1a shows a crossover event initiated by a double-strand break (DSB) on the right arm of chr IV in the first cell cycle after plating results in the white/red-sectored JSC25-1 colonies. Break-induced replication (BIR) would produce red/pink- or white/pink-sectored colonies (Fig. 1a). This means that the frequency of sectored colonies determines the degree of chromosomal instability. Our results showed that the cell viability of JSC25-1 was reduced to 86%, 28%, and 1.2% after heat treatment (52 °C) duration of 3 min, 3.5 min, and 4 min, respectively (Fig. 1b). Without heat shock, the frequency of white/red JSC25-1 colonies was 3 × 10^–5^ (Fig. 1c), and this rate was elevated 2, 4, and 11 times after heat shock for 3 min, 3.5 min, and 4 min, respectively (Fig. 1c). Using the PCR diagnosis described in the materials and methods section, we found that 2 of the 25 red parts of the sectored colonies came from the loss of YJM789-derived chr IV. By contrast, no chromosome loss was detected in the 25 spontaneous sectored colonies. These results suggest that heat shock can stimulate mitotic recombination and chromosome instability in yeast.

### Chr IV–specific SNP microarray analysis of sectored colonies

**Fig. 2:**
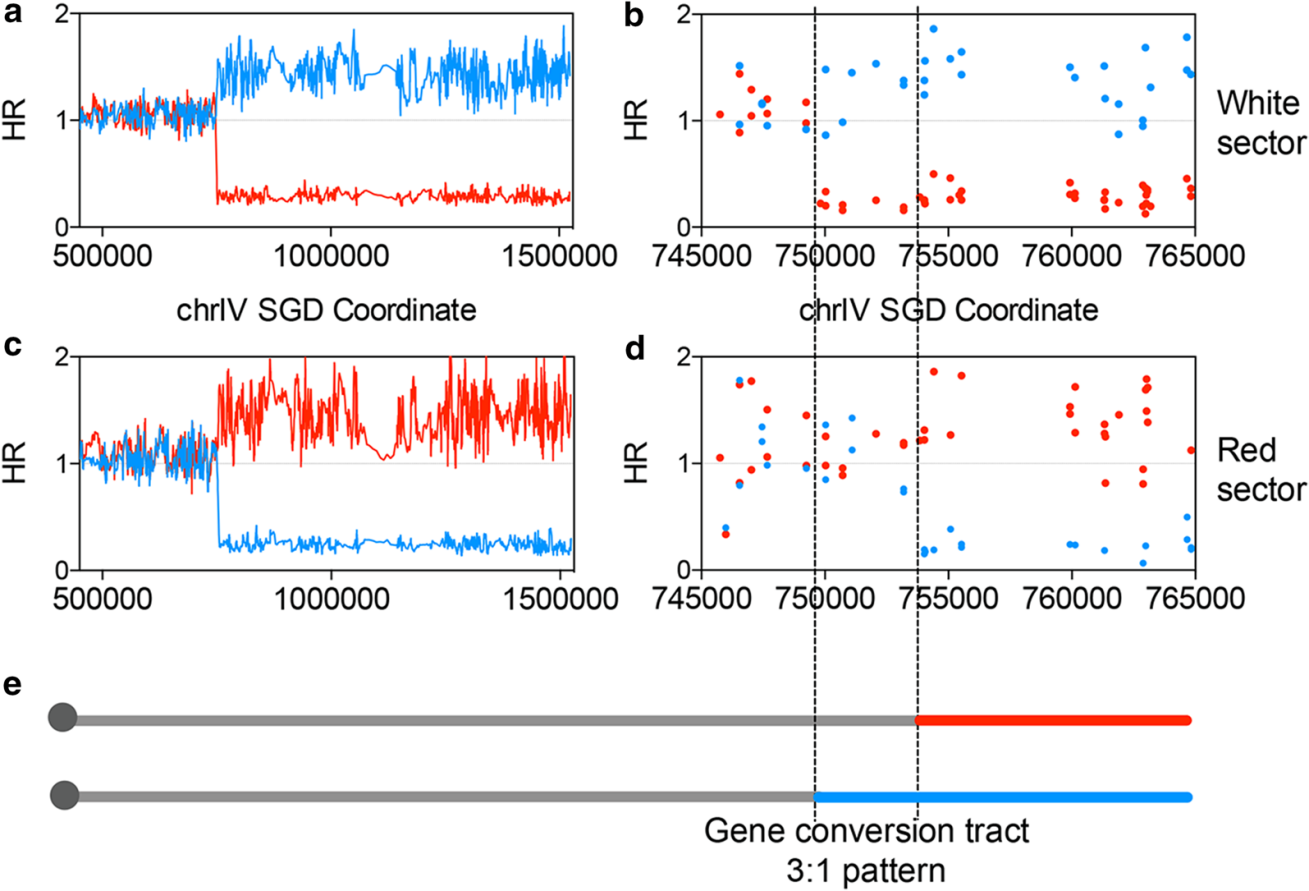
Analysis of crossover events by chr IV-specific SNP microarray. The Y-axis values show the normalized hybridization ratio (HR) of genomic DNA to oligonucleotides that are specific to W303-1A- and YJM789-derived SNPs. The X-axis values indicate the SGD coordinates of the SNPs. The hybridization ratio values of about 0.2, 1, 1.5 represent 0, 1, and 2 homolog copies, respectively. The red and blue lines/points indicate the HR of W303-1A- and YJM789-derived SNPs, respectively. The analysis results of the white sector and the red sector are shown in **a** and **b** at low resolution; the high-resolution results are shown in **c** and **d**. **e** The gene conversion tract pattern associated with this crossover event

To map the selected crossover events in the white/red-sectored colonies, genomic DNA was extracted from the white and red sectors, respectively. The sample DNA and control DNA were competitively hybridized on a customized SNP microarray specific to chr IV (1.1 Mb, accounting for up to 10% of yeast genome) (St Charles and Petes 2013). This array includes 2,300 SNPs across the right arm of chr IV, allowing for the mapping of crossover events to about 0.5 kb resolution (Additional file 1: Fig. S1). In addition, other genomic regions were represented by sparse SNPs that allows for the detection of all chromosomes’ aneuploidy events (Additional file 1: Fig. S1). Figure 2 shows one example of the SNP microarray results. Since a single mismatch (SNP site) is sufficient to destabilize short duplexes, the genomic DNA homozygous for the W303-1A-derived SNPs hybridizes better with the W303-1A-derived probes than with the YJM789-derived SNPs and vice versa. The blue and red lines/points in Fig. 2 indicate the hybridization level of the DNA sample from W303-1A-derived and YJM789-derived SNPs, respectively. The hybridization ratio (HR) for each oligonucleotide was normalized to the Cy5/Cy3 ratio of all the oligonucleotides on the microarray. HR values close to 1.5, 1, and 0.2 indicate 2, 1, and 0 copies of a homolog, respectively. In the white sector, the hybridization signal was transferred from heterozygosity to homozygosity (YJM789-derived homolog) between coordinates 749,244 and 750,033 (Fig. 2a, b). In the red sector, a signal transition was identified between 753,192 and 754,057 (Fig. 2c, d). For this crossover event, the region from 749,244 to 754,057 bp was identified as the crossover-associated gene conversion tract, which should include the initial recombinational lesion (Fig. 2e). Considering both sectors within the gene conversion tract, the blue SNPs were represented thrice, and the red SNPs were represented once (Fig. 2e), defined as a 3:1 pattern. As discussed in previous studies (St Charles and Petes 2013; Yin and Petes 2013; Zhang et al. 2019), such a pattern indicates that the initial recombinational lesion is likely to be a DSB occurring at one sister chromatid in the S/G_2_ phase (Additional file 1: Fig. S2A). If a DSB took place in the G_1_ phase, both sister chromatids would harbor DSBs at the same location after DNA replication (Additional file 1: Fig. S2B). DSB repair on both sister chromatids led to a 4:0 region within the gene conversion tract (Additional file 1: Fig. S2B). Of the 16 analyzed crossover events, 4 have no detectable conversion tracts, 7 have 3:1 pattern tracts, and 5 have complex patterns (containing 4:0 region). These results indicate that DSBs in the S/G_2_ phase initiated more than half of the heat-shock-induced crossover events. It should be noted that the 4 crossover events with no detectable conversion tracts were not included in this calculation, because the phases of their initial DSBs were not identified.

Besides the crossover events on the right arm of chr IV, we also observed 8 chromosomal aneuploidy events, demonstrating the tendency of heat shock to cause chromosome aberration (Additional file 1: Table S1). We found 3 monosomic chromosomes (chr III, chr IX, and chr VI) and 5 trisomic chromosomes (chr III, chr IX, chr VI, chr VIII, and chr XVI) (Additional file 1: Table S1). As shown in Additional file 1: Table S1, we observed the monosomy of YJM789-derived chr III in the red sector and the trisomy of YJM789-derived chr III in the white sector. Such a paired event took place in three sectored colonies. These results indicate that heat shock would interrupt the normal segregation of sister chromatids and cause chromosomal nondisjunction.

### Whole-genome SNP microarray analysis of JSC25-1-derived isolates after heat shock

To explore how heat shock affects chromosomal stability at the whole-genome level, JSC25-1 cells (2 OD_600_) were heated at 52 °C for 4 min and plated on YPD plates to form colonies. A total of 21 colonies (named JP1–JP21) were randomly selected for SNP microarray analysis.

### Mitotic recombination events and loss of heterozygosity (LOH)

**Fig. 3:**
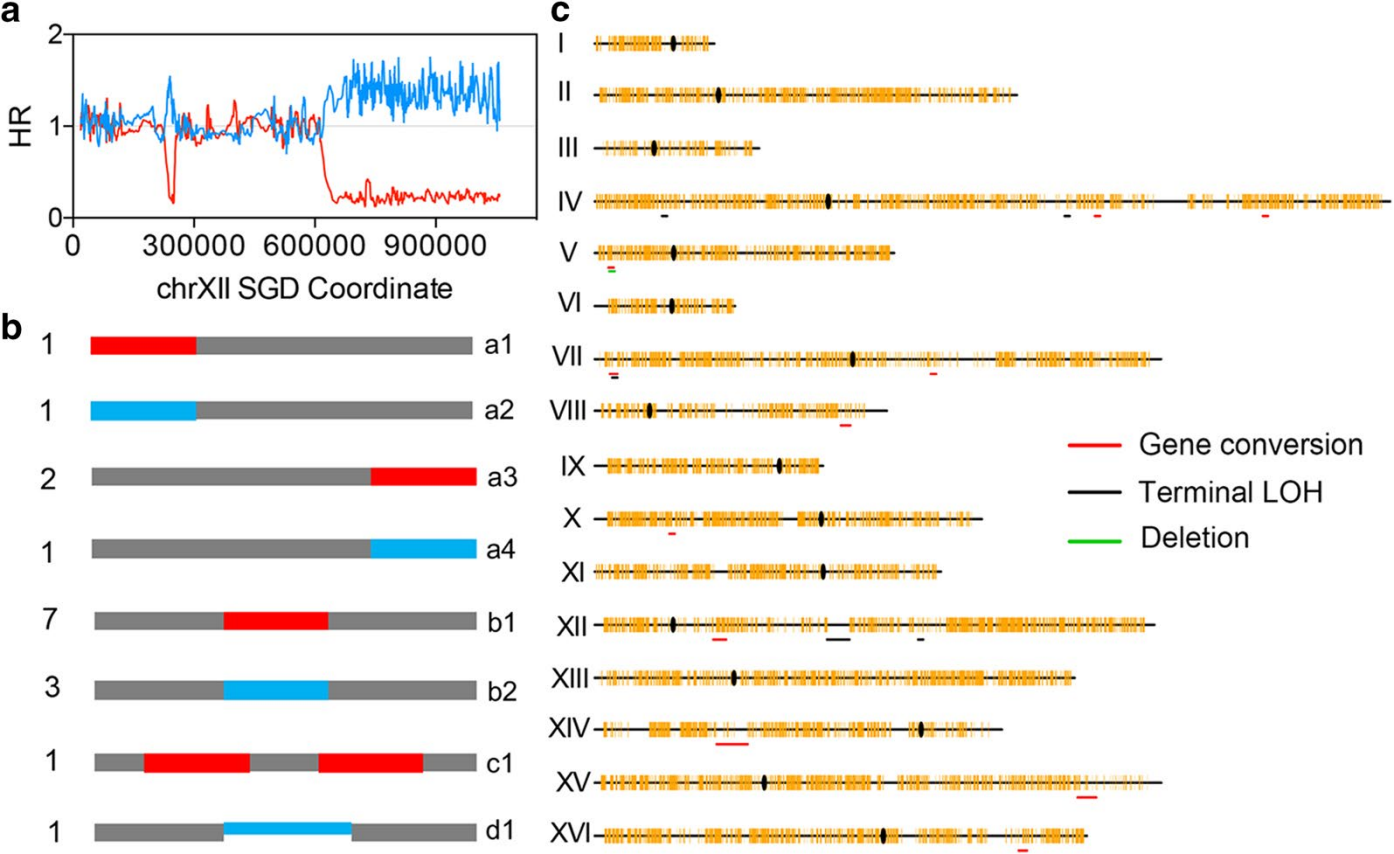
Whole-genome mapping of heat-shock-induced genomic alterations in the 21 JSC25-1-derived isolates treated with heat shock. **a** LOH events detected on chr X in the JSC25-1-derived isolate JP14. **b** Patterns of genetic events. The red and blue lines represent W303-1A- and YJM789-derived homologs, respectively. The gray lines indicate heterozygous regions. Classes a1–a4 represent terminal LOH events; b1, b2 and c1 represent gene conversion events; and d1 represents deletion event. **c** The genetic event distribution across the 16 chromosomes. The yellow vertical lines represent SNP sites

In yeast, mitotic recombination is the main pathway to repair DSBs during vegetative growth, which is crucial to cell viability in the presence of DNA damage agents but inevitably leads to LOH (Symington et al. 2014). Thus, detection of LOH events in the heat-shock-treated yeast cells allows the determination of heat-shock-induced DSBs and mitotic recombination. As shown in Fig. 3a, we observed an increase in the signal of YJM789-derived SNPs and a decrease in the signal derived from the W303-1A homolog near 240 kb of chr XII. This result indicates an internal LOH (gene conversion) on the chr XII, which may be explained by the repair of a DSB around 240 kb on the W303-1A-derived chr XII using the YJM789-derived homolog as a template. In addition, we also observed that the region from 625 kb to the right end was homozygous for the YJM789-derived sequence. Such terminal LOH might be due to a reciprocal crossover or a BIR event, as shown in Fig. 1a. In summary, we found 17 genomic alterations, including 11 gene conversions, 5 terminal LOH events, and 1 internal deletion in the 21 JSC25-1-derived isolates after heat shock (Fig. 3b and Additional file 1: Table S2). The rate of mitotic recombination in heat-shock-treated cells was calculated at about 3 × 10^–2^ events per genome per cell division (17 events/21 isolates/25 cell divisions) during the growth from a single cell to a colony on the YPD plate. Figure 3b shows the patterns of all detected genetic events, and Fig. 3c presents the distribution of genetic events across 16 chromosomes. All these genetic events have unique genomic locations, indicating that none of these events took place before heat shock. Previously, O’Connell et al. (2015) detected 10 LOH events in 10 diploid yeast isolates (isogenic to JSC25-1) that underwent 5,000 cell divisions, which means that the spontaneous rate of mitotic recombination in a wild type strain was about 2 × 10^–3^ per genome per division. Our results showed that heat shock (52 °C for 4 min) elevated the mitotic recombination rate by at least one order of magnitude at the whole-genome level.

### Aneuploidy events detected in the JSC25-1-derived isolates

**Fig. 4:**
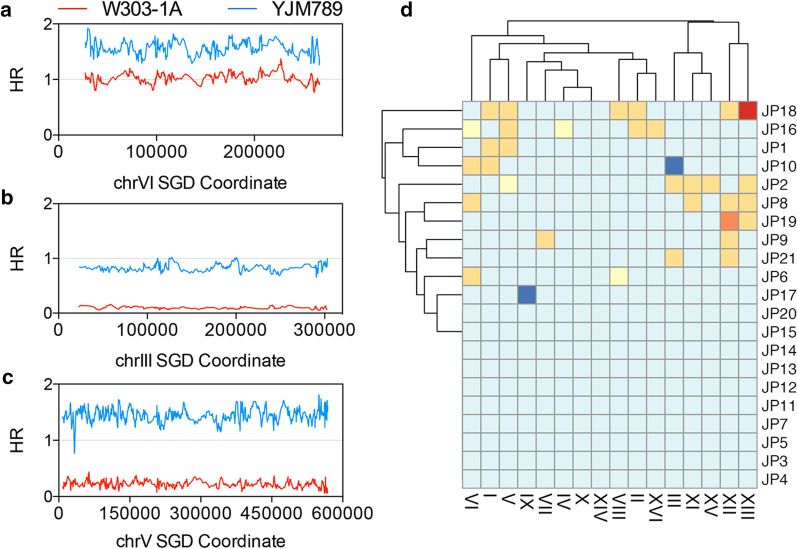
Aneuploidy events observed in the JSC25-1-derived isolates. The SNP results in **a**–**c** indicate trisomy, monosomy, and UPD event, respectively. **d** Heat-shock-induced aneuploidy events in the 21 JSC25-1-derived isolates. Red, orange, yellow, light yellow, blue, and dark blue represent pentasomy, tetrasomy, trisomy, uniparental disomy, normal, and monosomy, respectively

Besides mitotic recombination, aneuploidy events occurred frequently in the 21 heat-shock-treated, JSC25-1-derived isolates. Figure 4a, b show examples of chromosome loss (monosomy) and duplication (trisomy), respectively. In some cases, the HR values of one chromosome were reduced to 0.2 while those of the other were increased to 1.5 (Fig. 4c). This pattern indicates a uniparental disomy (UPD) event. In Fig. 4d, we showed chromosome copy number changes in the 21 isolates. There were 26 trisomic chromosomes, 4 uniparental chromosomes, 2 monosomic chromosomes, 1 pentasomic chromosome, and 1 tetrasomic chromosome. The aneuploidy event frequency was about 6.5 × 10^–2^ (34 events/21 isolates/25 cell divisions) in the heat-shock-treated cells. At a horizontal level, the isolates with similar aneuploidy events were clustered into groups. We found that 11 of the 21 isolates had at least one aneuploidy event. In addition, trisomic chromosomes occurred at a significantly higher rate than monosomic chromosomes in the heat-shock-treated yeast cells, which may be because chromosome loss is strongly and dominantly deleterious. Zhu et al. (2014) identified 29 trisomy and 2 monosomy events in 145 wild-type diploid isolates that underwent ~ 311,000 cell divisions, indicating that the spontaneous rate of aneuploidy is about 1 × 10^–4^ events per diploid genome per generation. Thus, our results showed an elevated chromosomal aberration rate of two orders of magnitude by heat shock.

### Different chromosomal instability patterns caused by heat shock and carbendazim

**Fig. 5:**
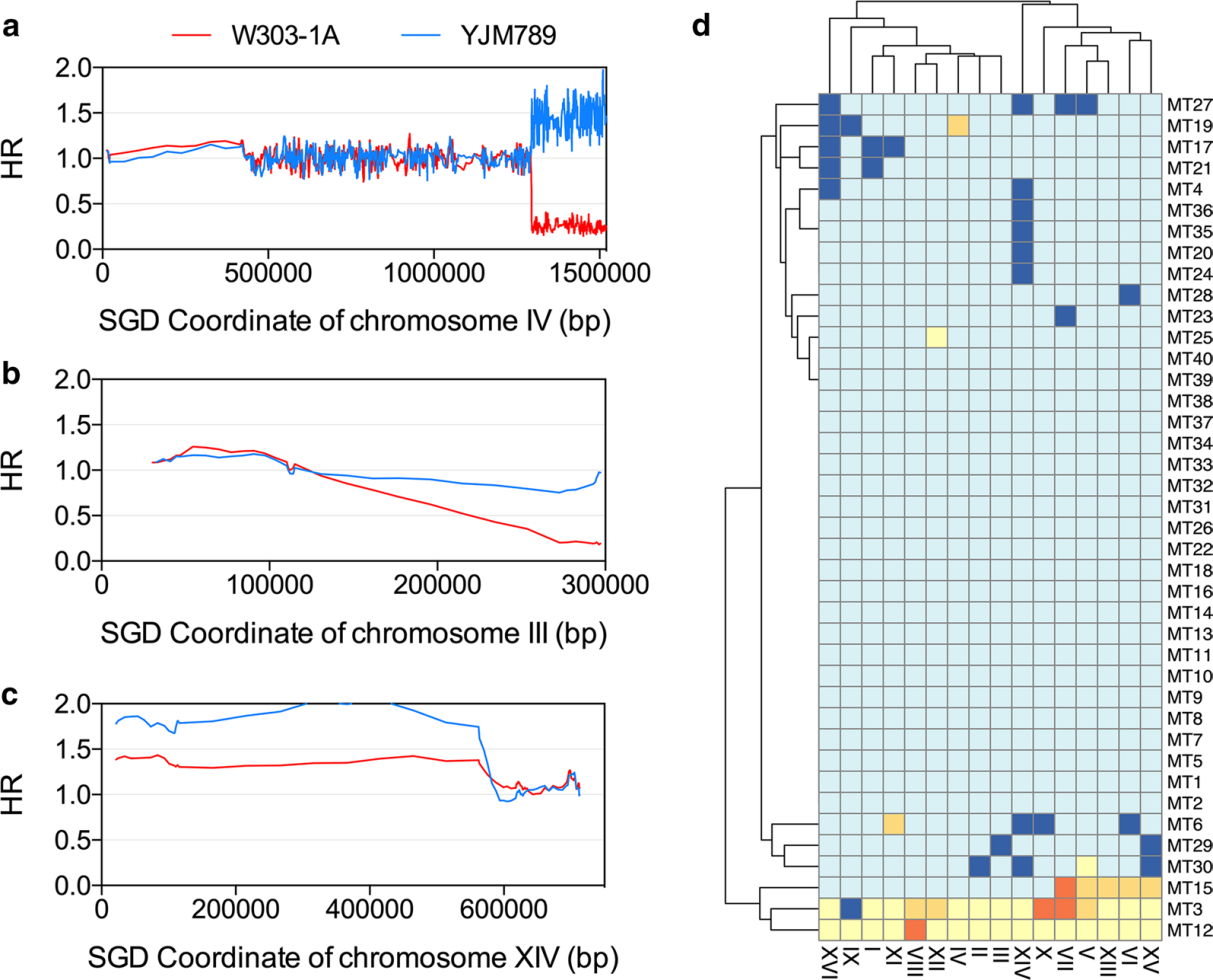
Chromosomal instability resulting from carbendazim treatment in JSC25-1. Genomic alterations were detected in 40 carbendazim-treated isolates (MT1–MT40) by the chr IV–specific SNP microarray. **a** An example of a carbendazim-induced terminal LOH event on the right arm of chr IV. **b** A terminal deletion event on the right arm of chr III. **c** A terminal LOH duplication event on the left arm of chr XIV. The genetic events in **b** and **c** were observed in the same isolate. **d** Aneuploidy events resulted from carbendazim treatment. Orange, yellow, light yellow, blue, and dark blue represent tetrasomy, trisomy, uniparental disomy, normal, and monosomy, respectively

We hypothesized that heat-shock-induced aneuploidy could be ascribed to the suppression of microtubule assembly dynamic. To test this hypothesis, chromosomal instability patterns caused by heat shock and carbendazim (microtubule inhibitor) were compared. Forty JSC25-1-derived isolates (MT1–MT40) were treated with 25 mg/L carbendazim for 2 h and then plated on YPD plates. For each isolate, only one colony was randomly selected for chr IV–specific SNP microarray analysis. In the 40 selected mutants, we observed 10 terminal LOH events (2 on chr II, 3 on chr IV, 3 on chr VII, 1 on chr X, and 1 on chr XVI). Figure 5a shows an example of these events. In the mutant MT39, we detected a deletion of the right arm of the W303-1A-derived chr III (Fig. 5b) and a duplication of the left arm of the YJM789-derived chr XIV (Fig. 5c). Such paired event is likely to reflect the DSB repair occurring on the right arm of chr III using the left arm of chr XIV through the BIR pathway. In Fig. 5c, we showed at least one aneuploidy event in 18 mutants. In the 40 analyzed mutants, there were 28 monosomic chromosomes, 9 trisomic chromosomes, 4 tetrasomic chromosomes, and 27 UPD (Fig. 5d). Interestingly, 25 of these 27 UPD events occurred in two mutants (MT3 and MT12). The peculiar karyotypes of these two isolates suggest that carbendazim treatment readily causes UPD. Except for MT3 and MT12, other isolates tended to lose chromosomes (Fig. 5c). This result was consistent with those of previous studies showing that carbendazim exposure causes frequent chromosome loss in both yeast and mammalian cells (Wood 1982; Zuelke and Perreault 1995; Zheng et al. 2017). Using Fisher’s exact test, we found that the monosomy–trisomy ratio in carbendazim-treated cells was significantly higher than that of heat-shock-treated cells. This result indicated various mechanisms underlying the carbendazim- and heat-shock-induced aneuploidy events.

### Heat shock drives phenotypic diversification in JSC25-1

**Fig. 6:**
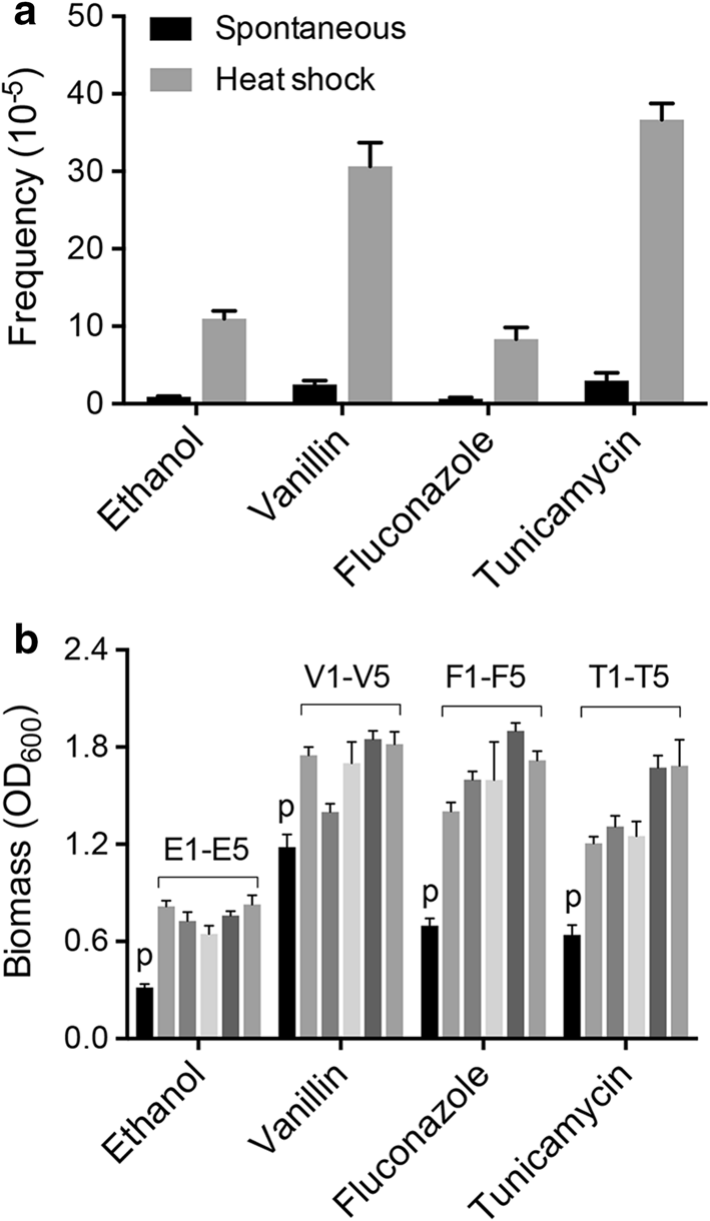
Heat shock drives fast phenotypic variations in yeast population. Experiments were performed three times, and the means are shown here. Error bars represent the standard deviation. **a** The frequency of resistant JSC25-1 isolates appeared on the solid YPD medium containing 120 g/L ethanol, 0.3 mg/L fluconazole, 1.2 g/L vanillin, and 4 mg/L tunicamycin. In this experiment, about 1 × 10^–4^ viable cells were plated on each plate. **b** Comparison of biomass formation (OD_600_) in yeast isolates in liquid YPD medium containing 70 g/L ethanol (E1–E5), 0.15 mg/L fluconazole (F1–F5), 0.8 g/L vanillin (V1–V5), and 1.5 mg/L tunicamycin (T1–T5). The OD_600_ of each sample was determined at the 12 h time point. The JSC25-1 parental strain is indicated by “p”

While chromosome aneuploidy and large-scale chromosomal rearrangements are always detrimental to mammalian cells, these genetic events enhance the adaptability of yeast under certain conditions (Gilchrist and Stelkens 2019). To examine whether and to what extent heat shock can promote phenotypic changes in yeast, we compared untreated and heat-shock-treated cells in terms of the frequencies of resistance mutants that can appear on the YPD plates with stressors. Compared with untreated cells, the cells treated with heat shock showed at least 10 times more resistant colonies on the YPD plate containing 120 g/L ethanol, 0.1 g/L fluconazole, 1.2 g/L vanillin, or 4 mg/L tunicamycin (Fig. 6a). Five independent isolates from different stressor-containing plates were purified on the YPD plates (from single cells to colonies). All the purified isolates from the YPD plates still showed better tolerance than that of the parental strain JSC25-1 under the test conditions (Fig. 6b), showing that heat-shock-induced phenotypic variations were not caused by a transient transcriptional or post-transcriptional state. Since ethanol-resistant strains have promising applications in bioethanol production, the five mutants selected from ethanol-containing plates were analyzed using whole-genome SNP microarray. Additional file 1: Table S3 shows the genetic events on the genomes of the five mutants. Interestingly, 5 of the 7 genetic events occurred on chr IV, including 1 UPD and 4 terminal LOH (Additional file 1: Table S3). As shown in Additional file 1: Table S3, all the five genetic events led to the homozygosity of large regions on the right arm of chr IV. A possible explanation for the improved ethanol tolerance of these five selected isolates will be discussed below.

## Discussion

A sudden temperature increase creates ubiquitous environmental stress for microorganisms in both natural and industrial conditions. This study explored the effects of heat shock on genomic integrity. Our main findings are that (1) heat shock can greatly simulate chromosomal instability in yeast, (2) more than half of the heat-shock-induced LOH were initiated by recombinational lesions in the S/G_2_ phase of the cell cycle, (3) chromosomal aberration was the most frequent genomic alteration in the heat-shock-treated yeast cells, (4) different chromosomal aberration patterns were observed between the heat-shock- and carbendazim-treated cells, and (5) heat shock drives phenotypic variations in yeast populations.

In the wild-type yeast cells, mitotic recombination is the main pathway responsible for LOH events and large-scale chromosomal rearrangements (Symington et al. 2014). We found that heat shock (52 °C for 4 min) elevated the crossover rate by about tenfold on the right arm of chr IV in yeast (Fig. 1). In agreement with this assay, in the whole-genome SNP microarray, we found that the rate of LOH events in the heat-shock-treated isolates was about 15 times higher than the spontaneous rate of LOH events (O’Connell et al. 2015). By analyzing gene conversion tract patterns associated with crossover in the sectored colonies, we found that about 60% resulted from the recombinational lesion repaired in the S/G_2_ phase. Previously, St. Charles and Petes showed that most (~ 70%) spontaneous reciprocal crossover events were initiated by DSBs in the G_1_ phase (St Charles and Petes 2013). In the heat-shock-treated cells, more recombinational lesions occurred in the S/G_2_ phase, suggesting that the genomic DNA in the S/G_2_ phase is more vulnerable to heat shock. This hypothesis was further supported by the observation of heat-shock-induced DSBs in the S phase of mammalian cells (Velichko et al. 2015). Based on the established homologous recombination theory, gene conversion events mainly reflect DSB repair through homologous recombination using the synthesis-dependent strand annealing pathway while terminal LOH could result from the double-strand break repair pathway or BIR pathway (Symington et al. 2014; Yim et al. 2014, 2017). The rate of gene conversion and terminal LOH event in heat-shock-treated cells was similar with that of spontaneous LOH event, indicating that the choice of repairing pathway was not greatly altered by heat shock.

After entering mitosis, the replicated genome is packaged into mitotic chromosomes, with each containing two identical sister chromatids. All chromosomes are then bioriented to allow for the segregation of sister chromatids toward the mitotic poles. Spindle assembly defect and many other factors would interrupt the partitioning of the two genomes and cause chromosomal distribution errors (Quevedo et al. 2012; Germann et al. 2014). Our results showed that heat shock can elevate the aneuploidy rate by hundreds of times. The frequency of aneuploidy events (6.3 × 10^–2^ per genome per division) was even higher than that of LOH events. By contrast, LOH events occur at a much higher rate than aneuploidy events in wild-type cells under normal conditions (O'Connell et al. 2015; Qi et al. 2019a, b). Interestingly, we found that heat shock produced more trisomic chromosomes than monosomic chromosomes in yeast, and the opposite result was found in carbendazim-treated cells (spindle assembly inhibition leads to failure in pulling the chromatid into cells). This comparison suggests that heat-shock-induced aneuploidy was not caused by spindle assembly defect. In the white/red-sectored colonies, we found that paired monosomy and trisomy occurred simultaneously in the first cell cycle after heat shock (Additional file 1: Table S1). This observation shows that heat shock would lead to a chromosomal segregation error in which both copies of the duplicated chromosome are deposited into one daughter cell and none into the other. One possible mechanism underlying heat-shock-induced chromosomal aberration is the unsuccessful cohesin breakdown, which opposes the splitting force exerted by microtubules. Although chromosomal nondisjunction results in an equal chance of trisomy and monosomy, the skewed trisomy–monosomy event ratio in heat-treated cells might be explained by the fact that trisomic chromosomes always have less of an impact on growth under normal conditions than monosomic strains (Gilchrist and Stelkens 2019). Alternatively, outnumbered trisomy events might be caused by re-replication of chromosome. It was reported that heat stress led to origin re-firing and centrosome amplification in the S phase of human HeLa cells (Petrova et al. 2016). In summary, our results demonstrated that both heat shock and carbendazim are potent inducers of chromosomal aberration. Considering different patterns can result from heat shock and carbendazim treatment, the combination of these two methods may generate more complicated karyotypes in yeast.

Aneuploidy, large-scale chromosomal rearrangements, and LOH are ubiquitous genomic alterations in solid tumor cells, as well as in yeast population (Zhu et al. 2016; Heil et al. 2017; Sansregret and Swanton 2017; Cho and Jinks-Robertson 2019; Gilchrist and Stelkens 2019). Many studies suggest that these genetic events would promote the adaptive evolution of yeast cells in response to environmental stimulus or genetic perturbations (Tan et al. 2013; Gerstein et al. 2015; Heil et al. 2017; Hose et al. 2015; Zhang et al. 2017, 2019). Based on these findings, carbendazim treatment was used in our previous study to construct robust mutants with improved tolerance to vanillin, a common inhibitor in lignocellulosic hydrolytes (Zheng et al. 2017). Our current results suggest that heat shock can develop quick adaptability to inhibitors of ethanol fermentation and antifungal drugs. Using the whole-genome SNP microarray, we found that the right arm of chr IV in several ethanol resistant isolates became homozygous to the SNPs of YJM789. In W303-1A, the gene *SSD1* (1,045,640 to 1,049,392 on the right arm of chr IV), which encodes a translational repressor (an mRNA-binding protein that interacts with untranslated regions), is deactivated by a premature stop codon (Avrahami-Moyal et al. 2012). This mutation reduces competitive fitness and increases sensitivity to ethanol and high temperature (Avrahami-Moyal et al. 2012; Hose et al. 2020). Thus, it is highly probable that the genetic events (including both aneuploidy and crossover events) resulting in the homozygosity of YJM789-derived *SSD1* were positively selected after heat shock and contributed to the ethanol tolerance of yeast as well.

To the best of our knowledge, this study is the first to show data demonstrating that heat shock could significantly stimulate chromosomal instability at the whole-genome level and phenotypic diversification in yeast. We also provided a convenient and efficient method to generate aneuploidy yeast mutants with potential in basic research as well as in industrial application.

## Supplementary information

**Additional file 1.** Additional figures and tables.

## Data Availability

The SNP microarray raw data was then entered in a GEO database (https://www.ncbi.nlm.nih.gov/geo/) with accession numbers GSE112062 and GSE150711.
