# Supplementary material for: Heat shock drives genomic instability and phenotypic variations in yeast
Source: AMB Express. 2020 Aug 17;10:146. doi: 10.1186/s13568-020-01091-7 (PMC7431486; doi:10.1186/s13568-020-01091-7)
Supplement: Supplementary file 1 — Additional file 1. Additional figures and tables. [file 13568_2020_1091_MOESM1_ESM.pdf]

AMB Express

Heat shock drives genomic instability and phenotypic variations in yeast

Li Shen, Yu-Ting Wang, Xing-Xing Tang, Ke Zhang, Pin-Mei Wang, Yang Sui*, Dao-Qiong Zheng*

Ocean College, Zhejiang University, Zhoushan, Zhejiang Province, 316021, China

*Corresponding author. Tel: +86 571 88206636; Fax: +86 571 88206627; E-mail address: suiyang@zju.edu.cn (Y.S) or zhengdaoqiong@zju.edu.cn (D-Q.Z)

Table S1 Aneuploidy events in the 15 sectored colonies (S1-S15).

**Sectored colonies**	**Aneuploidy events**
S4	Monosomy of YJM789-derived chromosome III in the red sector and trisomy of YJM789-derived chromosome III in the white sector
S7	Monosomy of YJM789-derived chromosome IX in the red sector and trisomy of YJM789-derived chromosome IX in the white sector
S11	Trisomy of W303-1A derived chromosome VIII and XVI in the red sector
S15	Trisomy of W303-1A derived chromosome VI in the red sector and monosomy of W303-1A derived chromosome VI in the white sector

Table S2. Genomic alterations detected by whole-genome SNP microarray in 21 JSC25-derived isolates (JP1-JP21).

Isolate	Chr	Event[a]	Class	Markers flanking transitions		Coordinates of LOH event	
				Left	Right	Left	Right
JP1	V	CON	b1	30652	31251	30652	32231
				31251	32231		
JP4	IV	CON	b1	967022	967772	967022	969944
				969369	969944		
	XII	Ter	a4	447537	490725	447537	490725
JP6	V	Del	d1	32231	32429	32231	34235
				33770	34235		
	VII	CON	b2	647381	653149	647381	657746
				655388	657746		
	XVI	CON	b2	816528	824722	816528	832669
				831138	832669		
JP8	IV	CON	b1	1289953	1292325	1289953	1293979
				1292496	1293979		
	VII	CON	b1	29386	32836	29386	43381
				33882	43381		
JP9	VII	Ter	a2	33882	43381	33882	43381
JP11	VIII	CON	b1	474198	482764	474198	492456
				487802	492456		
JP13	IV	Ter	a1	132549	136505	132549	136505
	IV	Ter	a3	909432	910662	909432	910662
	X	CON	b1	144943	146303	144943	153061
				149069	153061		
	XIV	CON	b2	234905	247556	234905	294378
				268741	294378		
	XV	CON	c1	930009	931533	930009	966210
				935109	942300		
				949471	958063		
				958063	966210		
JP14	XII	CON	b1	228409	234166	228409	253263
				250968	253263		
	XII	Ter	a3	623970	631728	623970	631728

[a] CON, Ter, and Tel indicates gene conversion, terminal LOH, and deletion event, respectively.

Table S3. Genomic alterations detected by whole-genome SNP microarray in ethanol tolerant mutants E1-E5.

Isolate	Chr	Event[a]	Class	Markers flanking transitions		Coordinates of LOH event	
				Left	Right	Left	Right
E1	IV	Ter	a4	793712	793805	793712	793805
E2	IV	Ter	a4	1111969	1142607	1111969	1142607
	XII	UPD					
E3	IV	Ter	a4	749244	750033	749244	750033
E4	IV	Ter	a4	471247	476888	471247	476888
	XVI	CON	b1				
E5	IV	UPD					

[a] CON, Ter, and UPD indicates gene conversion, terminal LOH, and uniparental disomy, respectively.

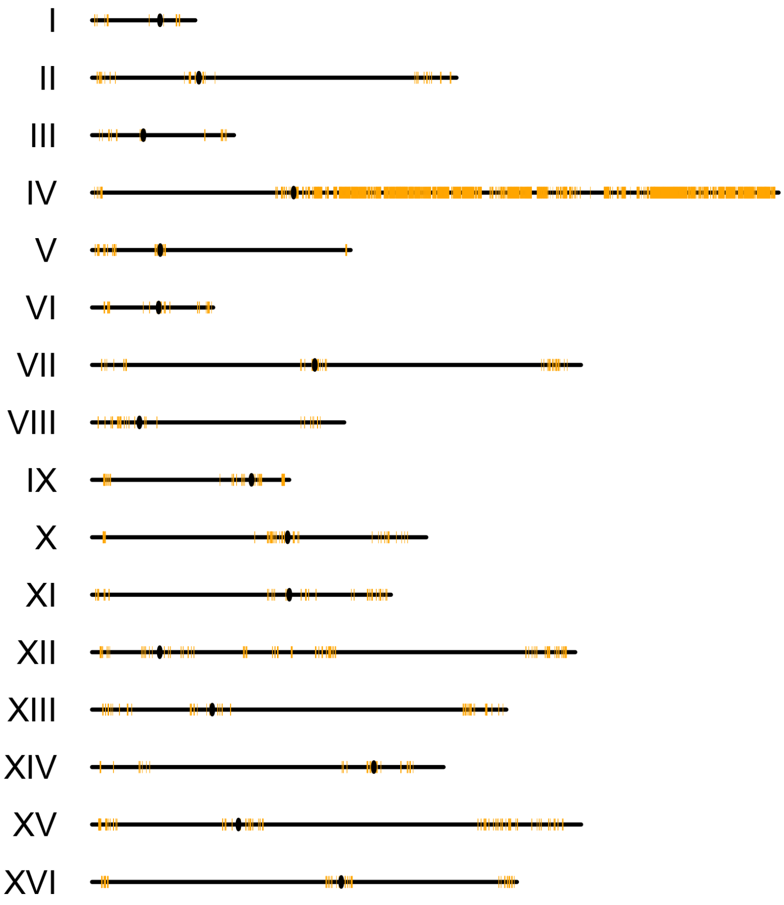

Fig. S1. The distribution of SNP markers across 16 chromosomes (I-XVI) of yeast genome of the custom SNP microarray. Centromeres are shown as black ovals and SNPs as short yellow vertical lines.

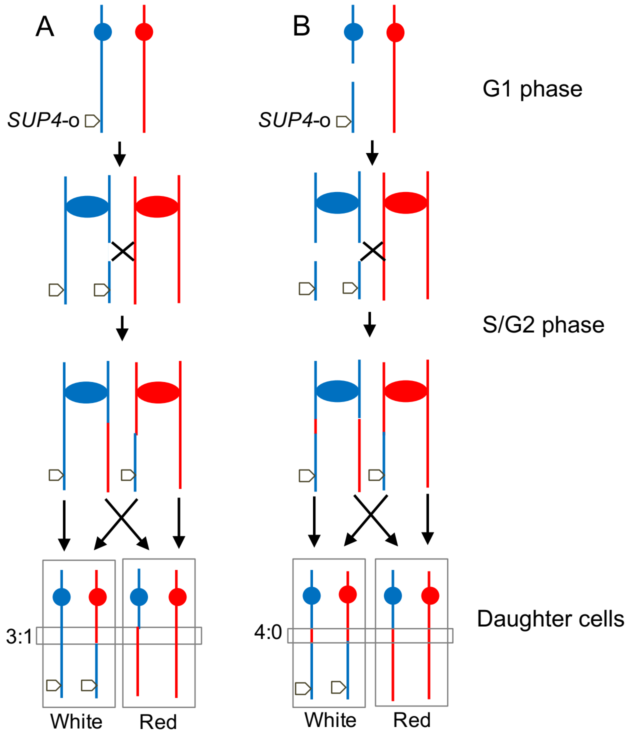

Fig. S2. Different patterns of gene conversion tracts generated by crossover events initiated by the double strand breaks occurred on (A) S/G2 phase and (B) G1 phase. The blue and read lines represent YJM789- and W303-1A-derived homologs in the diploid strain JSC25-1.
